# Synchronized RF and HIFES Technology for Clinically Significant Wrinkle Reduction: A Multicenter 3D Evaluation

**DOI:** 10.1111/jocd.70394

**Published:** 2025-08-13

**Authors:** Chris W. Robb, Joel L. Cohen, Amanda Holden, J. D. McCoy, Lesley Clark‐Loeser, Jennifer Levine

**Affiliations:** ^1^ Skin & Allergy Center Spring Hill Tennessee USA; ^2^ AboutSkin Dermatology and AboutSkin Research Greenwood Village Colorado USA; ^3^ Holden Timeless Beauty San Marcos California USA; ^4^ Contour Medical Gilbert Arizona USA; ^5^ Precision Skin Institute Davie Florida USA; ^6^ JL Aesthetics New York New York USA

**Keywords:** HIFES, noninvasive, radiofrequency, wrinkle reduction antiaging

## Abstract

**Background:**

The pursuit of youthful skin reflects its association with health and well‐being. Energy‐based procedures are increasingly favored for their noninvasive nature and ability to target signs of aging at the tissue level through controlled thermal or mechanical stimulation.

**Aims:**

This study evaluated the HIFES combined with synchronized radiofrequency (RF) as a noninvasive wrinkle treatment.

**Methods:**

Thirty‐three patients underwent four treatments using HIFES and synchronized RF. Follow‐up visits occurred at 1 and 3 months post‐treatment. Wrinkle severity was assessed using 3D photographs and expressed as a percentage change, while 2D photographs were evaluated using the Global Aesthetic Improvement Scale (GAIS) and the Fitzpatrick Wrinkle and Elastosis Scale (FWES). Patient‐reported outcomes included a 5‐point Likert scale satisfaction survey and a comfort questionnaire.

**Results:**

Significant wrinkle reduction was observed. 3D analysis showed wrinkle improvements of 20.13% ± 0.33% post‐treatment, 23.15% ± 0.32% at 1 month, and 35.36% ± 3.34% at 3 months. At 3 months, 88% of participants improved by at least one GAIS point. FWES score declined from 5.6 to 3.8, reflecting a shift from wrinkle severity class II to I. High satisfaction and comfort were reported.

**Conclusions:**

HIFES combined with synchronized RF effectively reduces wrinkle severity, as evidenced by objective measurements and patient feedback. This noninvasive treatment offers a safe and well‐tolerated option for improving skin appearance and quality.

## 1 Introduction

Age and ultraviolet‐associated changes in the skin may manifest as dyspigmentation, textural irregularities, and rhytids [1, 2]. In addition to its biological role in immunity, defense, and control of homeostasis, the skin also has important roles in socialization. The skin is often the first sign of health, beauty, and well‐being to be perceived by individuals. Thus, the condition of one's skin carries psychosocial implications [3]. As a result, major innovations and efforts have been made to develop technology, cosmeceuticals, injections, and energy‐based procedures to help reverse skin aging. The global anti‐wrinkle market was assessed to have a value of approximately 92 billion US dollars in 2023, growing at an annual rate of around 10.1% [4]. Current modalities for wrinkle reduction range from topical treatments to invasive surgical procedures, each with its limitations and advantages [5].

Facial aging is a multifactorial process that manifests on micro and macro scales [6]. The molecular effects of aging often translate into visible morphological changes in the face [7]. The dermal extracellular matrix (ECM) contains collagen and elastin fibers as its major components, providing tensile strength and elasticity to the skin [8]. In aging, collagen undergoes significant quantitative and structural changes, leading to fragmented and amorphous fibrils, ultimately resulting in loss of elasticity, leading to solar elastosis, laxity, wrinkles, and skin texture alterations [9]. Age‐related degradation of the cutaneous microvasculature networks also contributes to facial aging [10]. The alteration in delivered nutrients reduces the antioxidant capabilities of the dermis, as well as the metabolism of fibroblasts [11]. On the macro scale, age‐related atrophy and reduction in tonicity of facial muscles, such as the zygomaticus musculature, contribute further to the laxity and descent of facial structures [12].

Contemporary strategies for combating milder wrinkles often prioritize treatments that enhance collagen production, other components of the extracellular matrix, and skin hydration, utilizing approaches such as topical serums and creams [13]. Alternatively, for more significant wrinkles, surgical or laser procedures such as face lifts or full resurfacing involve extended downtime and carry additional risk factors.

To reconcile the complexity of aging across the depths of various facial structures with a holistic antiaging approach, a device synchronizing monopolar RF and HIFES technology has been developed. RF technology has garnered attention as a promising noninvasive approach for reducing wrinkles by targeting key molecular processes within the skin's dermal layer [14]. The HIFES counterpart induces highly selective contraction of zygomaticus and frontalis musculature, effectively targeting the muscles to build myofibril networks and increase their resting tone [15]. By enhancing the muscle tone that supports the suspended cheek fat compartments, the natural dynamic of the facial layers can be lifted and tightened like a well‐worked hammock up to its original position [16]. The present study aimed to evaluate the HIFES and synchronized RF technology as a noninvasive modality for treating wrinkles. The combination of RF + HIFES technology offers a comprehensive approach to wrinkle reduction and skin revitalization by bolstering the skin's intrinsic processes of muscle tone and dermal strength.

## 2 Methods

Thirty‐three (33) subjects (27 women and 6 men, skin types II–VI, with an average age of 58.1 ± 13.4 years) were recruited across six study sites. During the enrollment process, a thorough evaluation of inclusion and exclusion criteria, along with a review of each participant's medical history, was conducted. The inclusion criteria comprised individuals aged 21 years and older, a comprehensive understanding of the investigational nature of the treatment, acknowledgment of potential benefits and side effects, the presence of clearly visible wrinkles in the designated treatment area, a commitment and capability to abstain from any facial treatments other than the study procedure throughout the study duration, and a willingness to adhere to study instructions, including returning to the clinic for required visits and allowing photographs of their face to be taken. Participants who met any of the exclusion criteria, such as having metal implants (like dental or surgical implants), local infections, or unhealed wounds in the treated area, were ineligible to participate in the study. Additionally, previous cosmetic procedures that may affect the results of the study, such as rhytidectomy, neuromodulators, and volumizing fillers 3 months prior to the study were regarded as exclusion criteria.

### 2.1 Ethical Consideration

This multicenter study included six sites and had a single‐arm, open‐label, interventional design; received approvals from the Advarra Institutional Review Board; and was conducted in accordance with the ethical guidelines outlined in the 1975 Declaration of Helsinki. Before the commencement of any study‐related procedures, all patients willingly signed the provided informed consent. To ensure confidentiality, each patient was assigned a distinctive subject identification number.

### 2.2 Treatment Protocol

All patients underwent treatments utilizing the EMFACE (BTL Industries Inc., Boston, MA) equipped with a combination of HIFES with synchronized Radiofrequency (RF) technology. The treatment phase encompassed four sessions, each lasting 20 min, with intervals of 5–10 days between sessions. The energies were concurrently applied to the forehead and both cheeks using single‐use adhesive applicators. Before each therapy session, the treatment area was cleansed of any cosmetics, lotions, jewelry, and facial hair. During each session, the intensity of HIFES and RF stimulation was customized based on the patient's feedback. All patients were required to complete the full regimen of four treatments and attend two follow‐up visits at the 1‐ and 3‐month mark (with a permissible deviation of +/− 10 days). Patients were continuously monitored and examined for potential adverse events throughout the study.

### 2.3 Data Collection and Evaluation

Two standardized systems were utilized to thoroughly assess wrinkles at the study sites, depending on the available equipment. The LifeViz Mini, a three‐dimensional photographic imaging system developed by QuantifiCare S.A., France, was employed to capture facial images of patients undergoing evaluation. Both at baseline and during the 1‐ and 3‐month follow‐up visits, two‐dimensional photographs were captured from different angles, encompassing left, right, and frontal views of the face. These images were subsequently integrated into a 3D model utilizing QuantifiCare's software suite. The 3D models were assessed for wrinkle severity by considering the depth, length, and width of wrinkles in the treated areas, taking into account each subject's age, gender, and skin type. Each analysis received a score within a range of‐10 to +10. A negative score indicated that the wrinkle severity was worse than average, while positive scores above 0 denoted the extent to which the patient's results surpassed those of an average individual with similar age, gender, and skin type.

In parallel, high‐resolution 3D images of participants' faces were captured from multiple angles using the Vectra H2 (Canfield Scientific Inc., US) cameras. The accompanying software subsequently processed the acquired images to generate comprehensive 3D models of facial features. Wrinkle detection involved the software's algorithms analyzing skin texture and depth to identify areas with wrinkles, which were then quantified and assigned a numerical score ranging from 0 to 100 based on their severity. These scores were visualized on the 3D models, enabling clinicians to objectively evaluate participants' facial characteristics for treatment planning and outcome assessment. Data obtained from both 3D models were pooled and expressed as a percentage change. 3D photos were taken at baseline, after the last treatment, 1‐month, and 3‐month follow‐ups.

A 5‐point Likert scale Subject Satisfaction Questionnaire (SSQ) was administered following the final treatment and during all follow‐up visits to assess patient satisfaction with the therapy results. Additionally, after the conclusion of the final treatment session, a Therapy Comfort Questionnaire (TCQ) was administered, which included the Visual Analog Scale (VAS) with a range from 0 (no pain) to 10 (maximum bearable pain), along with a 5‐point Likert scale.

The Global Aesthetic Improvement Scale (GAIS) and Fitzpatrick Wrinkle and Elastosis Scale (FWES) were used to assess treatment outcomes for wrinkles based on 2D digital photographs. The GAIS, a widely accepted subjective assessment tool, was utilized to gauge overall aesthetic improvement post‐treatment. Participants' treatment outcomes were categorized into predetermined levels of improvement, spanning from “very much improved” (3) to “worse” (−1). Additionally, the FWES, a validated scale designed explicitly for evaluating facial wrinkles and skin elasticity, was utilized to assess wrinkle severity and skin firmness quantitatively. Three independent and trained evaluators conducted assessments based on predefined criteria, assigning scores to treated facial regions. Photographs were taken at baseline, after treatment, and at the 1‐month and 3‐month follow‐ups, with GAIS evaluated from post‐treatment, 1‐month, and 3‐month photos, and FWES evaluated from photos taken at all time points.

### 2.4 Statistical Analysis

Statistical analysis was conducted using the GraphPad Prism software (GraphPad Software, Boston, MA). Descriptive statistics, including the mean and standard error of the mean, were calculated. To ensure the compatibility of data obtained from two different imaging software programs, a statistical analysis was performed. For comparing two independent groups, the multiple Mann–Whitney test was used. To account for multiple comparisons, the Holm‐Sidak correction was applied to adjust *p* values. As the analysis showed no statistically significant difference between Vectra H2 and QuantifiCare, the results were merged. Merged data were assessed for normality using the Shapiro–Wilk test. The significance was assessed using the Friedman test, with *α* = 0.05 considered the significance level.

## 3 Results

**FIGURE 1:**
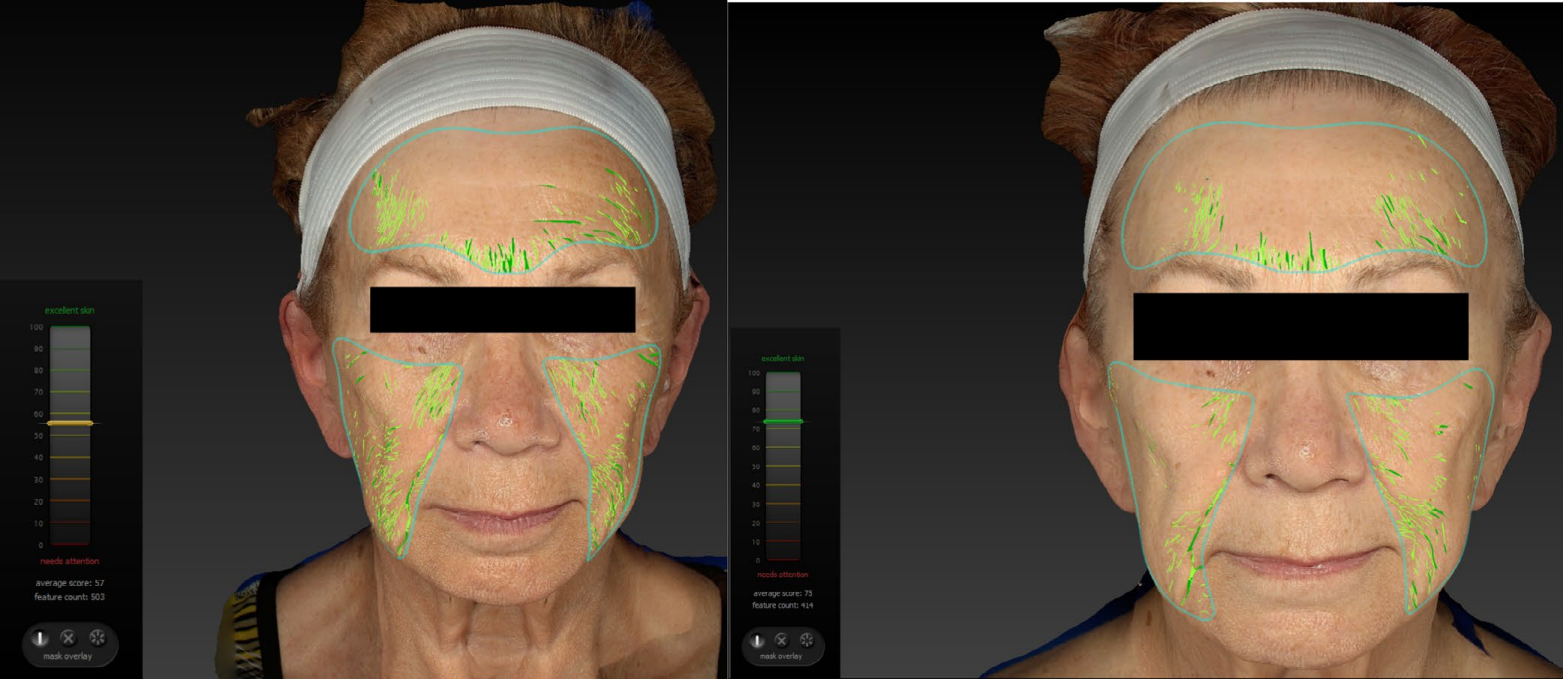
3D analysis of a patient at the baseline (left) and the 3‐month follow‐up visit (right) displaying a decrease in wrinkles denoted by the increase in score (left corner) from 57 at the baseline to 75 at the 3‐month follow‐up.

**FIGURE 2:**
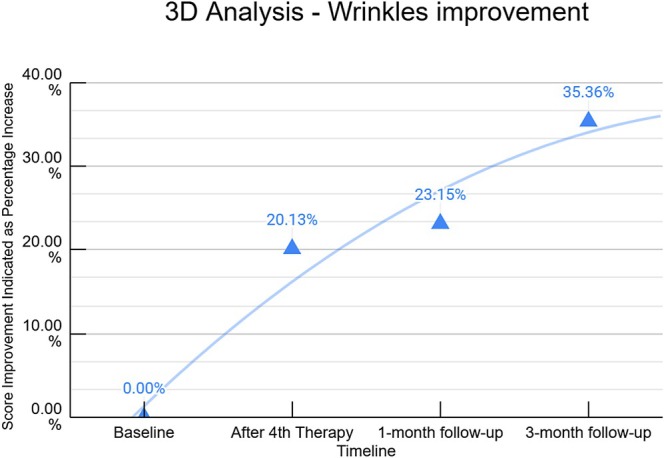
The Average Wrinkle Score, represented as percentage improvements acquired from 3D data. It shows a gradual increase throughout the study period, beginning at the baseline and ending at the 3‐month follow‐up.

The study included 33 participants (27 females and 6 males), with a mean age of 58.1 ± 13.4 years, all of whom successfully completed the study without the occurrence of adverse events. Out of 33 participants, 21 patients had 3D data available. Three‐dimensional evaluations revealed significant (*p* < 0.0001) improvements in wrinkle reduction post‐treatment (Figure 1). On average, 3D data analysis indicates that the patients showed an incremental improvement (*p* < 0.05) in wrinkles score by 20.13% ± 0.33%, 23.15% ± 0.32%, and 35.36% ± 3.34% (Figure 2) immediately after the last therapy, at the 1‐month follow‐up, and the 3‐month follow‐up, respectively.

**FIGURE 3:**
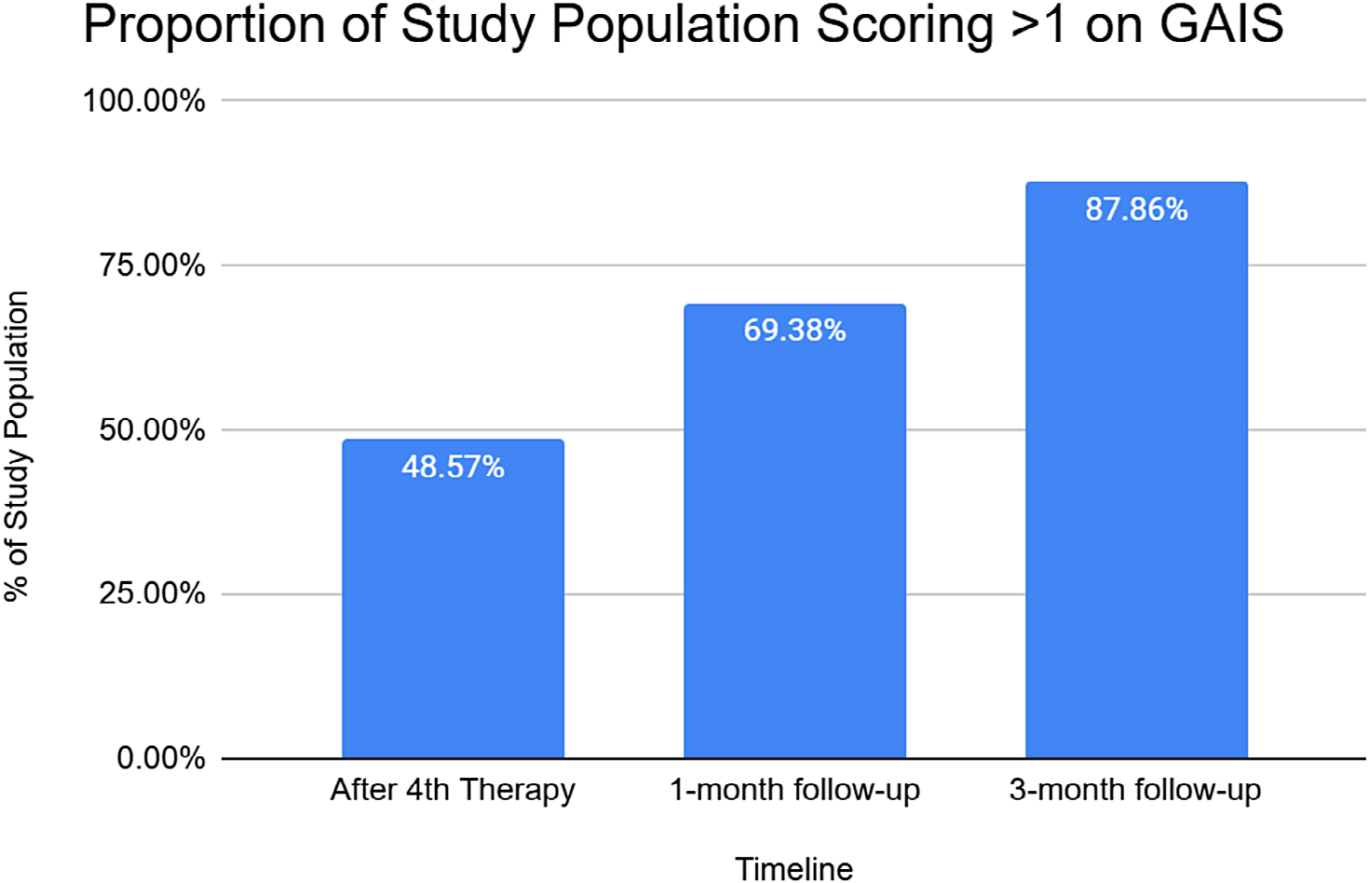
Graphical representation of the study population proportion reaching at least 1 point increase on the GAIS scoring throughout the study period, from the last therapy and ending at the 3‐month follow‐up.

**FIGURE 4:**
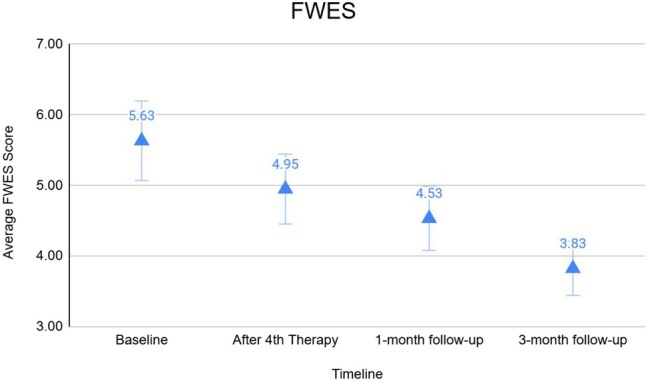
Graphical representation of the average FWES score decrease across the duration of the study period, beginning at the baseline and ending at the 3‐month follow‐up.

The 2D photographs of all participants encompassed within the study were accessible for analysis. GAIS scores demonstrated a notable enhancement in overall facial improvement, with 88% of participants scoring at least 1 point above the baseline by the 3‐month follow‐up (Figure 3). Assessments using the FWES exhibited a 35% improvement in wrinkle reduction among the study population at the 3‐month follow‐up (Figure 4). A strong correlation between the FWES and the data obtained through 3D analysis regarding wrinkle reduction was found (Pearson's *⍴* = 0.97). Additionally, the overall FWES score of 5.6 decreased to 3.8, indicating a shift in overall wrinkle severity from Class II to Class I.

The majority of patients (84%) agreed that the therapy was comfortable, while the other 16% neither agreed nor disagreed. A low average VAS score of 1.1 ± 1.8 was observed among all patients (*n* = 33). There were no negative responses from the SSQ evaluation immediately after the treatment, with the SSQ continuously rising at the 1‐month and 3‐month follow‐up, averaging at 86.8% of patients expressing satisfaction.

## 4 Discussion

The study evaluated the effects of HIFES and synchronized RF technology on wrinkles and rhytid reduction. All patients completed the study without adverse effects. The utilization of 3D evaluation techniques provided a comprehensive and objective assessment of the treatment's efficacy in wrinkle reduction and allowed for analysis of changes in wrinkle depth, volume, and distribution following treatment. The significant improvements observed in parameters such as wrinkle depth underscore the effectiveness of HIFES + RF treatment in achieving 35.36% ± 3.34% wrinkle reduction. Though two different imaging technologies were used (Vectra and Quantificare) for 3D modeling, the results were analogous. A similar trend in the FWES score further validates the consistency in results (Pearson's *ρ* = 0.91). FWES allowed for a standardized and systematic evaluation of wrinkle severity and appearance. Additionally, the overall FWES score of 5.6 has been lowered to 3.8, indicating a shift in overall wrinkle severity class, from Class II to Class I. The consistent and favorable reductions in FWES scores across the study population corroborate the positive impact of HIFES + RF treatment on wrinkle reduction and overall skin quality.

The favorable wrinkle reduction was achieved due to the combination of the delivered energy. Over the years, studies have shown that RF‐induced heating elevates tissue temperature, promoting metabolic activity and supporting collagen and elastin production [17]. In a synergistic manner, both RF and induced muscle contractions promote dermal microcirculation, leading to improved delivery of oxygen and nutrients to the skin cells and fibroblasts; supporting proposed heightened metabolic demands [18, 19]. The increased muscle tone not only provides better support for the overlying dermal and adipose tissue, thus diminishing wrinkles caused by sagging or laxity, but also exerts a gentle lifting effect, reducing the visibility of fine lines and wrinkles, especially around areas prone to dynamic expression [20, 21]. These processes induce innate physiological responses in order to achieve rejuvenation, in contrast to fillers or surgical interventions. Naturally, this implies reasonable room for improvement in the achieved cosmetic results as it provides a foundation for further cosmetic improvement. The zygomaticus musculature is not a targeted muscle when using neuromodulators, due to that approach having caused lateral lip immobility, asymmetry, and facial drooping [22]. Hence, integrating dermal fillers and nonproblematic zones of neuromodulators (such as glabella, crow's feet, DAO, peri‐oral muscle columns, etc.) with synchronized RF and HIFES could provide a superior holistic approach in facial rejuvenation, as well as allowing for fewer injection sites and thus lower risk of associated complications [15]. This proposed combination is supported by existing literature, including studies that have examined the safety and effectiveness of synchronized monopolar RF and HIFES in patients treated with botulinum toxin, as well as review articles discussing multimodal strategies for noninvasive facial rejuvenation [15, 23].

The study has utilized several objective methods when measuring the device's efficacy in reducing wrinkles. Analysis of data obtained through 3D‐rendered models of patients allowed for accurate quantification of the improvement regarding wrinkles. Future studies may benefit from longer follow‐ups and larger sample groups.

## 5 Conclusion

The study results of four consecutive treatments with a novel device combining HIFES and synchronized RF technology, administered once a week, have demonstrated high efficacy and patient satisfaction. Validated by 3D analysis, FWES and GAIS scales, in addition to patient comfort and satisfaction questionnaires, the modality reduces the appearance of wrinkles by 36%.

## Author Contributions

All authors have read and approved the final manuscript. C.W.R., J.L.C., A.H., J.D.M., L.C.‐L., and J.L. performed the research and analyzed the data. C.W.R. wrote the paper. J.L.C., A.H., J.D.M., L.C.‐L., and J.L. contributed to manuscript editing.

## Ethics Statement

The authors confirm that the ethical policies of the journal, as noted on the journal's author guidelines page, have been adhered to. All subjects voluntarily participated and signed a written informed consent. Additionally, informed consent was obtained from all participants for the use of their photographs.

## Conflicts of Interest

All authors have received payment or honoraria for lectures, presentations, speakers' bureaus, or educational events. These disclosures do not influence the integrity of the research presented.

## Data Availability

The data that support the findings of this study are available on request from the corresponding author. The data are not publicly available due to privacy or ethical restrictions.
